# From water to wards: status of integrated preventive strategies to break the chain of infection and antimicrobial resistance in Pakistan

**DOI:** 10.3389/fphar.2026.1783788

**Published:** 2026-03-25

**Authors:** Zikria Saleem

**Affiliations:** Department of Pharmacy Practice, College of Pharmacy, Qassim University, Qassim, Saudi Arabia

**Keywords:** antibiotic resistance, antibiotics, infection, preventive action, public health

## Abstract

**Background:**

Antimicrobial resistance (AMR) presents a serious and growing public health threat in Pakistan. To reduce risk of resistant infections, it is crucial to strengthen preventive efforts through improved water, sanitation, and hygiene (WASH) and robust infection prevention and control (IPC) measures.

**Methods:**

A narrative literature review along with document analysis methodology approach was chosen given the broad, heterogeneous nature of the topic, encompassing various intervention types and study designs.

**Results:**

The review identified a growing body of evidence on IPC and WASH in Pakistan, predominantly from urban centers after 2018. While most healthcare facilities fail to meet WHO IPC standards, targeted training and WASH-FIT (Facility Improvement Tool) initiatives have improved compliance and hygiene behaviors. Community-based programs such as handwashing promotion, improved water infrastructure, and education significantly reduced diarrheal diseases and antibiotic use, highlighting their potential role in mitigating antimicrobial resistance.

**Conclusion:**

Despite pockets of progress, Pakistan’s current preventive efforts are insufficient and limited to few settings contain AMR. Strengthening WASH infrastructure, enforcing IPC standards and instituting antimicrobial stewardship to reduce antibiotics prophylaxis are all urgently needed to break the chain of resistance from community water sources to hospital wards.

## Introduction

Antimicrobial resistance (AMR) has emerged as one of most pressing public health challenges (Mestrovic et al., 2025). In 2021, bacterial AMR was directly attributable for 1.14 million deaths and associated with 4.71 million deaths globally, with South Asia forecasted to remain a primary epicenter of this burden. Projections indicate these figures could rise to 1.91 million attributable and 8.22 million associated deaths annually by 2050 if preventive strategies are not scaled (Naghavi et al., 2024). Pakistan faces a perfect storm of factors fueling AMR: a high infectious disease burden, widespread antibiotic misuse, inadequate sanitation, and limited healthcare infrastructure (Saleem et al., 2022). The country ranks among the world’s largest consumers of antibiotics, and resistant “superbugs” such as extended-spectrum β-lactamase (ESBL) producing Enterobacteriaceae, methicillin-resistant *Staphylococcus aureus* (MRSA), and even colistin-resistant strains have been increasingly reported in both community and hospital settings (Arif et al., 2021; Klein et al., 2024). The implications are dire common infections are becoming difficult to treat, leading to prolonged illnesses, higher costs, and excess mortality.

Healthcare-associated infections (HAIs) negatively impact patients by increasing illness and death, extending hospital stays, and raising costs (Liu et al., 2025). Diarrheal illness acquired outside of a hospital, known as community-acquired infections (CAI), is transmitted through contaminated food or water and close contact due to poor hygiene. Viruses, especially rotavirus, adenovirus, and astrovirus, are the main culprits in most diagnosed cases (Chiejina and Samant, 2023). Up to 94% of diarrheal disease cases are linked to environmental factors and related risks, such as unsafe drinking water, low socioeconomic status, inadequate sanitation, and unhygienic habits like not washing hands before eating or after environmental activities. Other contributing factors include pathogen type, sociocultural customs, behaviors, and the accessibility and quality of health systems. Despite the availability of effective interventions like improved hygiene and sanitation, managing diarrheal diseases remains a significant public health challenge. Access to safe drinking water is vital for preventing diarrheal diseases, which present substantial public health issues, particularly in developing nations (Boafo et al., 2024).

To mitigate the burden of CAIs and HAIs, focused surveillance and preventive interventions including early infection screening and rigorous adherence to infection control protocols should prioritize high-risk populations (Parra et al., 2024; Tawfick et al., 2023). These infections significantly prolong hospital stays by an average of 8.3–11 days and increase direct medical expenditures by $2,037 to $9,807 per patient, with specialized cases like central line infections exceeding $45,000 (Zimlichman et al., 2013; Gidey et al., 2023; Jia et al., 2019; Moradi et al., 2024). Preventing infections in these patients can more effectively lessen the financial and duration-of-stay burdens associated with HAIs (Mwita et al., 2021). Implementing infection prevention and control (IPC) programs, HAI surveillance, proper medical staff training, and fostering a strong organizational culture can decrease the burden of HAIs. The World Health Organization (WHO) introduced the Infection Prevention and Control Assessment Framework (IPCAF) to assist countries and healthcare facilities in strengthening IPC practices. IPC programs offer evidence-based recommendations to prevent HAIs and combat antimicrobial resistance through effective IPC practices. These programs are mandated at both national and acute-facility levels (Parra et al., 2024). One crucial example of health attitudes, knowledge, and actions concerning personal and environmental hygiene that people should adopt is regular handwashing. Several elements influence handwashing behavior with soap, including awareness of its importance, knowledge of correct handwashing techniques, the availability of facilities and supplies, and the impact of social support within the community (Resmawati et al., 2025). Handwashing with soap is a fundamental and crucial method for preventing and controlling the spread of infectious diseases. A statistically significant link exists between handwashing with soap and the occurrence of diarrhea, as inadequate or incorrect handwashing contributes to diarrheal illnesses (Nisah et al., 2024). Various handwashing intervention methods can be utilized, such as mobile health-based interventions. A number of studies have been published on WASH (Water, Sanitation, and Hygiene) Health programs (Tidwell et al., 2019; Imaja et al., 2017; Henry, 2017). One study in India (Tidwell et al., 2019) reported an increase in handwashing with soap among mothers of young children following a 4- and 8-week mHealth program that used text message reminders for target WASH behaviors. Another study (Henry, 2017) in Tanzania aimed to promote handwashing with soap among youth through text messages, observing modest increases in handwashing behaviors. More evidence is needed on the global impact of WASH mHealth programs (Bhuyian et al., 2023).

Recognizing the threat, Pakistan released a National Action Plan (NAP) on AMR in 2017, aligned with the WHO Global Action Plan (Saleem et al., 2022). In 2023, the WHO’s Global Research Agenda on AMR in Human Health highlighted the critical need for evidence on preventive measures (Maryam et al., 2025). In Pakistan, however, the implementation of these strategies has lagged. Five years into the NAP, critical gaps persist between policy and practice (Saleem, 2025). Nevertheless, breaking the chain of infection and AMR transmission in Pakistan requires an integrated preventive strategy. In this context, an up-to-date review of Pakistan’s progress on integrated infection prevention strategies is warranted. This review examines the current status of preventive measures, spanning WASH, infection control, and other prophylactic interventions, aimed at containing infections in Pakistan. Ultimately, evidence-based insights were provided to inform policy and practice: what integrated approaches are working, what weaknesses must be addressed, and what recommendations can accelerate progress toward breaking the chain of infection and AMR transmission in Pakistan.

## Methods

A narrative literature review along with document analysis methodology approach was chosen given the broad, heterogeneous nature of the topic, encompassing various intervention types and study designs. This approach allowed inclusion of diverse sources from randomized trials of community interventions to cross-sectional surveys and surveillance reports of published research articles along with extensive review of grey literature of government documents, reports and working papers in order to map the current landscape of infection and AMR prevention. This methodology is specifically suited where evidence is fragmented across peer-reviewed journals and institutional policy documents. Unlike a conventional systematic review which focuses on a narrow clinical question, this hybrid approach emphasizes narrative synthesis to identify overarching themes and policy gaps. The rigor of this review is derived from the comprehensive inclusion of diverse data sources and a transparent document analysis of national guidelines, ensuring a holistic representation of the Pakistani healthcare context (Popay et al., 2006; Grant and Booth, 2009).

### Search strategy and selection criteria

A comprehensive literature search was performed across electronic databases (e.g., PubMed, Embase, Scopus) and local journals for published studies. Reference lists were also manually searched for relevant articles and gray literature or policy reports when pertinent (such as government or WHO or UNICEF reports). Search terms combined keywords for antimicrobials/resistance (e.g., “antibiotic resistance,” “drug-resistant,” “AMR”, “water sanitation,” “hand hygiene,” “vaccination,” “infection control”, “infections”) and relevant Mesh keywords with Boolean operators filtered for studies and reports pertaining to Pakistan. Studies were eligible if they met the following inclusion criteria: (1) addressed preventive strategies (community or hospital-based) to reduce infections; (2) conducted in Pakistan; (3) reported outcomes relevant to knowledge/behavior change. Interventional studies (randomized or quasi-experimental trials, implementation studies), observational studies (cross-sectional, cohort, qualitative) were included which provided data on practices or outcomes, and surveillance reports. Given the focus on human health, most included studies relate to human populations (community dwellers, patients, healthcare workers), though One Health aspects (e.g., environmental or veterinary reservoirs) were noted if directly relevant.

### Data extraction and synthesis

Using a standardized form, important data were extracted from each included study: citation details (author, year), study setting (geographic location in Pakistan; community vs. hospital), design and sample size, target population, duration, the specific intervention or focus (e.g., handwashing promotion, diagnostic test evaluation, resistance surveillance), the infection or disease area addressed (e.g., diarrhea, pneumonia, urinary tract infection), and main outcomes or findings (e.g., reduction in infection incidence, changes in antibiotic prescribing, resistance rates, *etc.*). For qualitative data, key themes and narratives relevant to infection prevention were extracted through thematic synthesis. For grey literature, historical, discourse, and semiotic analyses were conducted to trace policy evolution, interpret underlying narratives, and examine visual and linguistic representations of IPC and WASH within national health communication materials. Within each thematic category, findings from individual studies were summarized and compared in order to look for common trends as well as discrepancies. To enhance data interpretation, graphical representations were incorporated to illustrate key trends and patterns identified across studies. Quality appraisal of individual studies was not the primary focus due to the narrative nature. The synthesis instead aims to capture the breadth of evidence on what has been done and found regarding infection prevention in Pakistan. All data were double-checked for consistency against source publications.

## Results

**FIGURE 1:**
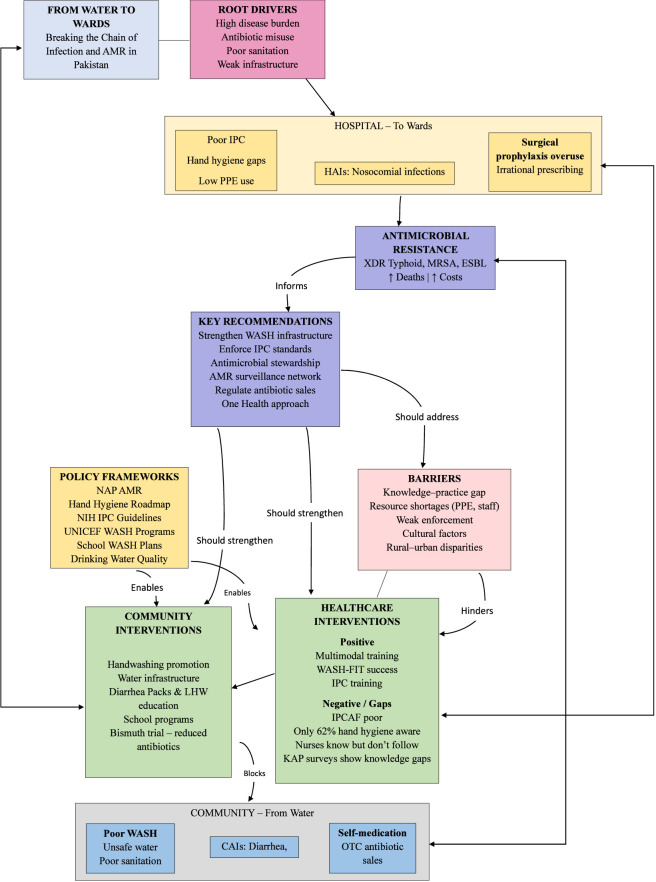
Conceptual flow diagram illustrating the connection of IPC, WASH with of AMR.

**FIGURE 2:**
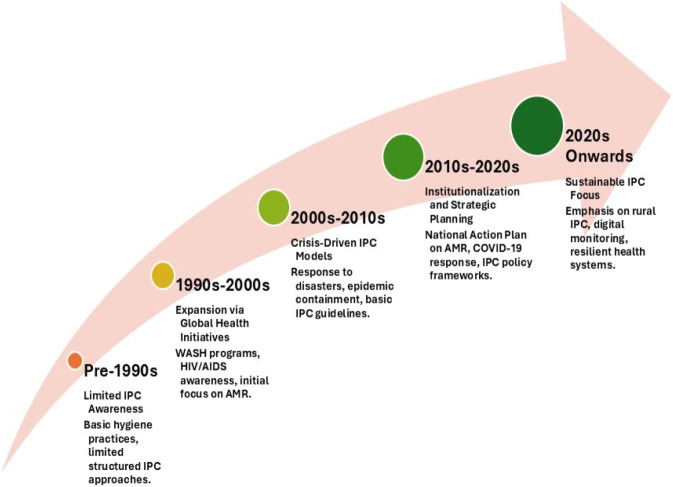
Historic analysis.

Our search identified relevant published studies and reports on infection prevention in Pakistan. These comprised community-based trials on WASH or related interventions, studies evaluating hospital IPC practices or interventions, and observational studies. The publications of these studies and reports noticeable accelerated in recent years: over half were published in 2018 or later, reflecting growing research attention to infection prevention following Pakistan’s NAP on AMR launch. The available evidence demonstrates a geographical concentration of studies in major urban centers of Punjab and Sindh provinces. The urban-centric evidence base in Punjab and Sindh creates “policy blind spots” that likely mask the true infection burden in rural regions like Balochistan and KP. To address this, national strategies must prioritize decentralized research funding, leverage digital health surveillance for remote areas, and mandate standardized provincial IPC/WASH audits. These measures are critical for developing a more equitable, impact-focused, and representative national policy framework. Figure 1 illustrates the conceptual linkage between IPC, WASH, and AMR and Figure 2 and Table 2 presents the historical progression and policy evolution of IPC and WASH initiatives in Pakistan.

Across Pakistan’s healthcare settings, IPC practices remain suboptimal, with most public hospitals failing to meet even basic WHO standards. WHO-IPCAF assessments showed that government hospitals in Islamabad and elsewhere scored far below global benchmarks, highlighting critical gaps in hand hygiene, availability of protective equipment, and staff training. A 2020 cross-sectional study at a Karachi tertiary hospital reported that over half of healthcare workers did not recognize hand hygiene as part of their role in preventing infections. In fact, only 62% were aware that improper hand hygiene could lead to nosocomial infections, while nearly 45% were unaware of such complications. Compliance is an even bigger issue: Batool et al. (2023) observed that although nurses in Quetta generally knew infection control protocols, in practice there were gaps (e.g., inconsistent use of masks or sterile technique), and the authors called for more awareness and training to improve adherence. Despite these challenges, targeted interventions have demonstrated effectiveness: structured training programs significantly improved hand hygiene compliance, safe sharps disposal, and PPE use among healthcare workers. Khan et al. (2022) implemented an intensive training program for auxiliary hospital staff in Punjab on hand hygiene, safe sharps disposal, and PPE use. After multimodal education, significant improvements were documented: hand hygiene compliance rose, proper sharps disposal practices improved, and PPE usage increased, compared to pre-training baseline. However, knowledge-practice gaps persist, many healthcare providers lack sufficient understanding of nosocomial infection risks, and routine adherence to IPC protocols remains inconsistent. Structured tools like WASH-FIT and intensive training initiatives have shown promise in raising facility-level IPC compliance, but systemic limitations such as lack of oversight, resource disparities between private and public hospitals, and absence of regulatory enforcement continue to impede progress.

In community settings, WASH interventions have yielded significant reductions in infection rates, particularly diarrheal diseases among children. Trials in Karachi and Gilgit demonstrated that simple handwashing promotion and improved water infrastructure reduced diarrheal incidence by up to 64%, indirectly lowering reliance on antibiotics. Luby et al. (2004a) conducted a community trial in Karachi’s squatter settlements where households received intensive handwashing promotion with soap; the intervention led to a 53% reduction in diarrheal illness in children under 5 (compared to controls). Integrated community programs such as the Diarrhea Pack and supportive supervision of Lady Health Workers successfully shifted care from inappropriate antibiotic use to evidence-based management using oral rehydrated solution (ORS) and zinc. These interventions not only improved hygiene behaviors but also strengthened frontline health workers’ capacity for rational prescribing. While direct measurement of AMR outcomes remains limited, reductions in infectious burden and improved hygiene practices suggest a meaningful contribution to AMR mitigation. Community health workers delivering clean water treatment and hygiene education have likewise improved household practices (e.g., more families boiling or filtering drinking water). School-based WASH efforts (improved latrines, drinking-water stations and hygiene education) also report rising knowledge and handwashing among pupils, though formal impact data are limited. Overall, these programs often run by NGOs, UNICEF or *via* the Lady Health Worker network have led to demonstrable reductions in infection risk factors (and thus likely antibiotic need) in target communities Details are given in Tables 1,3.

**TABLE 1:** Published literature.

Author name/Year/References	Location	Study design	Sample size	Population	Settings	Study duration	Target disease	Method	Outcome
Asghar et al. (2025)	Pakistan	Nationwide Cross sectional survey	42	IPC team	Hospital	NA	HAI	IPCAF questionnaire	Private hospitals outperformed public sector in IPC
Kiddeer et al. (2025)	Punjab	Cross sectional study	533	Healthcare personnel	Hospital	May-July 2019	HAI	KAP regarding standard precautions in tertiary care hospitals	Inadequate KAP; improved *via* certified training
Tunio et al. (2025)	Pakistan	Interventional Study	All	Orphanage School Children	Community	NA	CAI	Impact of handwashing practices on bacterial load	Hygiene programs strengthened health and education
Batool et al. (2023)	Quetta	Cross sectional study	138	Nurses	Hospital	NA	HAI	Preventive measures like hand hygiene, use of masks and apron, sterilized instrument	High IPC compliance and awareness
Khan et al. (2022)	Rawalpindi	Quasi experimental study	138	Auxillary Healthcare staff	Hospital	Aug-September 2020	HAI	Multimodal health education intervention addressing hand hygiene, safe sharp disposals, use of PPE	Training improved both IPC knowledge and compliance
Sehar et al. (2022)	Bagh	Quasi experimental study	32	Healthcare providers	Hospital	March-May 2020	HAI	WASH-FIT	Improved hygiene and waste management
Khaliq et al. (2022)	Karachi	Cross sectional study	384	Children and their mother	Community	Nov 2016-May 2017	Diarrhea	Measure relation between preventive measure knowledge and diarrhea	Integrated practices reduced diarrhea in urban slums
Zia et al. (2022)	Lahore	Cross sectional study	383	Healthcare worker	Hospital	Sep 2019-August 2020	HAI	Perception of hand hygiene	Knowledge unrelated to hygiene practice; infrastructure needed
Savul et al. (2021)	Islamabad	Cross sectional study	353	Healthcare workers	Hospital	January-March 2020	HAI	Assess satisfaction of IPC trainees and improvement of their knowledge and skills	Workshops boosted healthcare worker IPC skills significantly
Rabbani et al. (2021)	Mirpur khas	Qualitative study	22/16	LHSs, LHW, Community caregiver	Community	In 2017 and 2019	Diarrhea and pneumonia	Supportive supervision intervention	Improved skills and trust
Qureshi et al. (2021)	Karachi	Retrospective study	175/305	Children	Community	June 2017-December 2018	Diarrhea	Treatment outcomes with and without antibiotic regimen	Antibiotic use linked to longer hospital stays
Ahmed et al. (2020)	Karachi	Cross sectional study	212	Healthcare worker	Hospital	One month	HAI	Measure association between hand hygiene awareness and infection complication	Multidimensional approach needed for hand hygiene compliance
Akram et al. (2020)	Rawalpindi	Cross sectional study	780	Healthcare provider	Hospital	Feb-May 2020	HAI	Knowledge of HAI prevention by hand hygiene	Surveillance and behavioral change improved hygiene compliance
Savul et al. (2020)	Islamabad	Cross sectional study	5	Administrators	Hospital	Nov-December 2019	HAI	IPCAF tool in hospitals	Inadequate IPC; requires guidelines and supplies
Bowen et al. (2019)	Karachi	Randomized clinical trial	439/479	Outpatient clinics	Community	Aug-September 2015	Diarrhea	Compare effects of BSS and placebo	BSS use significantly reduced antibiotic consumption
Habib et al. (2013)	Jehlum	Randomized trial	26,000	Physicians, quack practitioner, healthcare provider, children and their mother	Community	June-August 2010	Diarrhea	Effectiveness of diarrhea packs	Reduced antibiotic use
Luby et al. (2006)	Central Karachi	Randomized control trial	6,962	General population	Community	April-December 2003	Diarrhea	Hand washing with soap	Home-based interventions are effective
Rasheed et al. (2005)	Karachi	Cross sectional study	8	Administrators	Hospital	NA	HAI	Assess the techniques and policies for safe disposal of waste	Rigorous enforcement of waste management guidelines
Luby et al. (2004a)	Karachi	Randomized control trial	36	Children	Community	15 April 2002-5 April 2003	Diarrhea	Hand washing with soap	Improved handwashing reduced diarrhea
Luby et al. (2004b)	Karachi	Interventional Study	228/130	Households	Community	May-October 2000 June-October 2001	Diarrhea	Impact of home-based interventions like soap, dilute bleach, water storage vessel	Wealthier households benefited sooner from hygiene interventions
Nanan et al. (2003)	Gilgit	Case control study	454/349	Children and their mother	Community	July –September 2001	Diarrhea	Assess effectiveness of WASEP	Integrated approach optimized hygiene and health benefits
Luby et al. (2001)	Karachi	Interventional Study	58/75	Manzoor colony	Community	NA	CAI	Measure hand cleanliness by providing soap, improving wash-water quality	Soap effective even with contaminated water

BSS: bismuth subsalicylate, CAI: community acquired infections, FIT: facility improvement tool, HAI: Healthcare Associated Infection, HOPE: Health-Oriented Preventive Education, IPC: infection prevention and control, IPCAF: infection prevention and control assessment framework, KAP: knowledge, attitudes and practices, LHSs: Lady Health Supervisors, LHW: lady health worker, NA: not available, WASEP: water and sanitation extension program, WASH: water, Sanitation, and Hygiene.

**TABLE 2:** Grey literature.

Document title	Year	Publisher	Key themes/Findings	Recommendations/Implications
UNICEF PATS Program Overview	2018–2022	UNICEF Pakistan	Describes UNICEF’s efforts to end open defecation and improve water access in Pakistan	Encourages more robust policy support for PATS initiatives
Water, Sanitation & Hygiene (WASH) in Public Sector Schools Strategic Plan for Sindh	2017–2022	UNICEF Pakistan	Schools lack adequate WASH infrastructure, impacting students’ health and learning; need for IPC integration in school curricula	Improve access to and usage of safe drinking water, sanitation, and hygiene facilities in schools by 2022
Hand hygiene Pakistan Roadmap	2021	Ministry of Climate Change	A country wide approach to achieving Sustainable universal hand hygiene	promote and sustain hand hygiene in Pakistan, particularly in the context of the COVID-19 pandemic
Drinking Water Quality in Pakistan: Current Status and Challenges	2021	Pakistan Council of Research in Water Resources	Access to adequate and safe drinking water	Raise awareness in the country
Urban wash Behavioural Determinants	2020	UNICEF Pakistan	Understand the social, cultural, economic, and psychological factors that influence how people use WASH services	Use this knowledge to design effective and sustainable strategies
WASH Sector Status Report	2019	UNICEF Pakistan	Reviews WASH status in Punjab, identifies critical gaps in sanitation and water quality	Highlights need for improved sanitation facilities and water quality in public spaces
Punjab Water, Sanitation, Hygiene Report	2019	Punjab Housing Urban Development and Public Health Engineering Development	Detailed assessment of hygiene practices in Punjab; poor sanitation impacts health outcomes	Calls for community-wide awareness programs and infrastructure improvements
National Guidelines COVID-19 and PPE	2020	WHO and NIH Pakistan	Guidance on Rational Selection & Use of Personal Protective Equipment	Reducing risk of acquiring COVID-19 infection in healthcare workers specifically and public at large
National Guidelines Infection Prevention & Control	2020	NIH Pakistan	Curtail infections and their spread in healthcare facilities	Improving the quality of services regarding IPC in healthcare facilities
School Health Education Guidebook	2018	WaterAid, Pakistan	Focuses on teaching hygiene practices in schools, emphasizing handwashing and sanitation	Suggests integrating IPC in school curricula to promote health education for children
Pakistan Occupational Health & Safety Act	2018	Govt of Pakistan	Provides IPC guidelines for workplace environments, especially industrial settings	Recommends adopting IPC practices across all workplaces
Addressing the Drinking Water Challenge in Pakistan	2018	Consumer Rights Commission of Pakistan	Consume water unfit for human consumption leading to water emergency	Formulation of National Water Policy
WaterAid School WASH Research Report	2016	WaterAid, Pakistan	Evaluates WASH infrastructure in schools, highlighting inadequate facilities	Calls for improvements in WASH facilities in schools to prevent hygiene-related diseases
Minimum service delivery standards	2012	Healthcare department, Punjab Government	IPC	Improve hospital standards
Pakistan safe drinking Water and hygiene Promotion project Final report	2010	USAID/Pakistan	Poor water quality is a major health risk; WASH programs aim to improve drinking water standards and accessibility	Calls for investment in water infrastructure, regular quality monitoring, and public awareness on safe drinking water practices
National Guidelines Infection Control	2006	NIH Pakistan	Curtail infections and their spread in healthcare facilities	Improving the quality of services regarding IPC in healthcare facilities

**TABLE 3:** Discourse analysis.

Key aspects and narratives	Actors & stakeholders	Underlying assumptions and ideologies	Implications
Perception of IPC as Essential Practice	General Public, Healthcare professionals	Post-pandemic, there is a heightened awareness of hygiene and mask-wearing, but practices are often not sustained once immediate threats are perceived as over	Points to the need for sustained public health education to normalize hygiene as a routine preventive measure
Public Health Emergency Preparedness	Government agencies, NGOs, healthcare providers	IPC practices are framed as reactive rather than proactive, emphasizing a response to crises	Encourages short-term IPC efforts but may overlook long-term structural improvements in IPC.
Community Empowerment and Education	Schools, local community leaders, NGOs	Assumes that knowledge leads to behavior change; communities will adopt IPC if educated about hygiene	Focus on education may neglect infrastructure needs or resource limitations that hinder IPC.
Professional Responsibility in Healthcare	Medical Students and Trainees, Healthcare professionals, policymakers	Indicates a gap in medical education but the responsibility for IPC lies heavily with healthcare professionals. Many healthcare workers experience increased anxiety and a greater responsibility regarding infection risks, especially post-COVID-19	May increase pressure on healthcare workers without adequate institutional support or resources
Environmental and Occupational Safety	Employers, employees, health authorities	Focuses on individual and organizational accountability, assuming that structured policies can address all risks	Underplays broader environmental factors affecting IPC, such as urban sanitation and infrastructure
WASH as a Developmental Imperative	UNICEF, WaterAid, government bodies	Positions WASH as essential for development, assuming economic growth will follow improved WASH.	Strongly promotes investment in WASH but may lack a nuanced approach for vulnerable populations
Antimicrobial Resistance (AMR) as a Global Threat	WHO, international health organizations	Framed as a global security threat, implying an urgent need for global coordination and rapid technological solutions	Pushes for quick solutions, potentially sidelining local and preventative IPC strategies
Resource Allocation and Policy Advocacy	Policymakers, international donors	Suggests that IPC challenges stem from insufficient funding and resource distribution rather than systemic inefficiencies	May lead to policy emphasis on funding without addressing systemic, structural, and managerial improvements
Rural-Urban Disparities in IPC	Rural communities, NGOs, local governments	Assumes rural communities are inherently disadvantaged and will benefit from urban-model IPC frameworks. As compared to urban areas lack of access to PPE, sanitation materials, and clean water leads to frustration and a sense of neglect in rural areas	Ignores context-specific solutions for rural areas and might impose unsuitable urban-centric models on rural communities
Policy and Enforcement Challenges	Policymakers	Policymakers experience difficulty in enforcing IPC guidelines consistently across healthcare facilities due to resource limitations and resistance	Highlights the disconnect between policy development and practical implementation, especially in under-resourced areas
Challenges with Behavioral Change and Emotional Impact of Outbreaks	Health Educators, Patients and Family Members	Patients and families report fear and anxiety during infectious disease outbreaks, especially regarding hospital-acquired infections	Emphasizes the need for transparent communication and visible IPC practices to reassure patients and families
Financial Burden of IPC Supplies	Healthcare Facilities	Hospitals and clinics, especially in low-income areas, report financial strain in maintaining consistent stocks of PPE and hygiene materials	Demonstrates the need for government and donor support to sustain IPC supplies, especially in resource-limited settings
View on Global Standards and Influence	Health Officials and Donors	Health officials see IPC policies influenced by global standards as necessary but sometimes feel they lack alignment with local realities	Suggests the need for a more locally adapted IPC model that considers Pakistan’s unique healthcare context

Grey literature from national and international agencies highlights critical gaps in Pakistan’s WASH and IPC systems, especially in schools, healthcare, and public institutions. Key documents from UNICEF, NIH, and WaterAid emphasize poor sanitation infrastructure, lack of hygiene education, and inconsistent PPE access across settings. Several initiatives such as the Pakistan Hand Hygiene Roadmap and the WASH Strategic Plan for Schools stress the need for IPC integration in curricula, public awareness, and sustainable hygiene practices post-COVID-19. National guidelines also point to weak enforcement mechanisms, suggesting that while frameworks exist, implementation remains fragmented. Details are given in Tables 2–4.

**TABLE 4:** Semiotic analysis.

Element	Sign	Signifier	Signified meaning	Implications
Handwashing Symbols	Icons showing hands with water droplets or soap	Visuals in IPC posters and pamphlets	Symbolizes cleanliness, hygiene, and the prevention of disease transmission	Reinforces the importance of hand hygiene; critical for IPC adherence in healthcare and public settings
Face Mask Imagery	Images of face masks or people wearing masks	Common in COVID-19 and IPC campaign materials	Represents protection from airborne pathogens, responsibility toward others	Encourages public compliance with mask-wearing; signifies care and community health protection
Color Coding	Use of green, blue, or red in IPC materials	Green for cleanliness, blue for healthcare, red for warnings	Green signifies safety, blue denotes health, red warns about contamination or risk	Color-coded messages create intuitive understanding, helping people quickly assess risks and take precautions
Water Symbolism	Images of clean water (faucets, water drops)	Featured in posters on sanitation and hygiene	Signifies purity, hygiene, and the foundation of health	Emphasizes the role of clean water in IPC; encourages water conservation and access to safe water sources
PPE Gear Icons	Icons or images of gloves, gowns, and goggles	Shown in healthcare settings and IPC guidelines	Denotes protection, safety, and preventive measures in healthcare	Visual reminder of the equipment needed to protect both healthcare workers and patients from infections
Soap and Sanitizer Icons	Bottles of soap or hand sanitizer on posters	Found in hand hygiene promotion materials	Signifies easy hygiene practices; encourages the regular use of soap/sanitizer	Reinforces hand hygiene as a routine habit for infection control across different environments
Bacteria/Virus Imagery	Microscopic images or stylized drawings of bacteria/virus particles	Often on educational posters and IPC materials	Represents the invisible threat, germs that cause infections	Visualizes the unseen danger, making the threat more concrete and the need for IPC more compelling
Slogans and Phrases	Phrases like “Stop the Spread,” “Clean Hands Save Lives”	Common in posters, training materials	Signifies urgency, personal responsibility, and the importance of hygiene practices	Simplifies IPC messages; memorable phrases increase retention and adherence to infection prevention
Government and WHO Logos	Logos of national health bodies, WHO, and other health organizations	Seen on official IPC documents and posters	Symbolizes authority, reliability, and global standards in IPC	Adds credibility to IPC messages, reinforcing public trust and encouraging adherence to guidelines
Educational Diagrams	Step-by-step diagrams for handwashing, PPE donning/doffing	Included in instructional IPC materials	Provides clear, sequential guidance for effective IPC practices	Improves comprehension of IPC practices; visual instructions cater to a broad audience, including low-literacy groups
Community Imagery	Pictures of families, children, and community groups practicing hygiene	Featured in IPC campaigns for public engagement	Represents collective responsibility, community health, and mutual protection	Promotes a sense of communal duty; reinforces the idea that IPC benefits the entire community
Hazard Symbols	Exclamation marks, skulls, or biohazard signs	Found on posters addressing hazardous waste and contamination risks	Signifies danger, contamination, and the need for caution	Enhances awareness of specific high-risk areas or actions, promoting safer behaviors around potential hazards
Cultural Symbols	Images and symbols that resonate with local culture, like traditional attire in health promotion visuals	Seen in community-specific IPC campaigns	Connects IPC practices with local cultural identity and values	Enhances relatability and acceptance of IPC practices by aligning them with cultural familiarity

## Discussion

Pakistan’s IPC situation closely parallels many other low- and middle-income countries (LMICs). WHO surveys show a clear income gap in IPC: high-income countries (HICs) overwhelmingly meet core IPC requirements, whereas LMICs (including Pakistan) lag far behind (Organization, 2022a). In the global facility survey, only ∼15% of LMIC hospitals met basic IPC standards *versus* >90% in HICs (Organization, 2022a). Similarly, studies in neighboring Bangladesh found that ∼73% of tertiary hospitals were at only a *basic* IPC level (median IPCAF ∼355/800) during the COVID-19 period (Harun et al., 2022). Like Pakistan, those hospitals often had isolation guidelines and some handwash stations, but no robust surveillance or regular training. In contrast, many Western hospitals have advanced IPC teams, routine audits and full compliance with WHO’s multimodal strategy. Thus Pakistan’s situation, major gaps in training, monitoring and facility hygiene, is consistent with broader LMIC trends. Notably, despite these challenges, the nationwide Pakistani survey found some “intermediate” IPC level (median IPCAF ∼388), suggesting pockets of progress; this is comparable to findings from Iran and other upper-middle-income settings. The data showing poor compliance with IPC standards and routine multi-day surgical prophylaxis reflect not just knowledge gaps but ingrained practices and systemic inertia (Saleem et al., 2023). Factors such as high patient loads, resource shortages (e.g., erratic supply of gloves or disinfectants in some public hospitals), and lack of accountability all play a role.

In Pakistan, a critical barrier is the knowledge–practice gap. Healthcare workers’ knowledge, attitude and perception (KAP) surveys reveal that even well-studied guidelines are not fully followed (Kiddeer et al., 2025). Cultural and workload factors contribute: hand hygiene compliance in Pakistani studies was often very low, even when wash stations were present. Changing these behaviours requires more than information (Resmawati et al., 2025). Global evidence indicates that multimodal interventions including leadership buy-in, feedback loops and routine audits are needed, but such systems are weak in Pakistan (Tahir et al., 2023). For instance, the Bangladesh study reported that 90% of hospitals lacked any IPC monitoring or feedback mechanism; presumably, similar deficiencies occur in Pakistan (Harun et al., 2022). International partnerships (e.g., with JCI or ICN) could be utilized to upskill infection control nurses and link Pakistani hospitals into global IPC networks.

Policy and health system support are equally crucial. Pakistan has published IPC guidelines and a National AMR Action Plan (2017), but experts note a disconnect between policy and practice (Saleem, 2025). Regulatory enforcement is weak, and antibiotic prescribing guidelines are outdated or ignored. By comparison, many HICs have both mandatory IPC programmes and antibiotic stewardship tied into accreditation. WHO and UNICEF global frameworks (Core IPC components, WASH in healthcare, “Three-Star” school hygiene) provide models, and Pakistan has begun to adopt these, e.g., national hand hygiene roadmap linking to global WASH targets (Organization, 2022b). Yet implementation remains spotty: funding constraints, chronic understaffing and lack of IPC focal persons undermine these efforts (Jafree et al., 2022).

The COVID-19 pandemic was a double-edged sword for IPC. On one hand, it dramatically raised awareness. Surveys showed that Pakistanis adopted key hygienic behaviours (e.g., handwashing, masking) during 2020–21 (ul Haq et al., 2021). In healthcare, demand for hand sanitizer and soap soared, and many hospitals enhanced cleanliness protocols. However, the sustainability of these changes is uncertain. The national Hand Hygiene for All roadmap explicitly warns that gains may not last once the crisis wanes. Indeed, studies stress that without continued reinforcement, handwashing rates can revert to pre-pandemic baselines. COVID also strained resources: while it prompted temporary IPC investments, it also led to excessive empiric antibiotic use for suspected COVID cases, fueling AMR risk (Hackman et al., 2024; Nandi et al., 2023).

The WHO Global Action Plan on AMR and Sustainable Development Goals both call for WASH and IPC as foundational to infection control (Lozano et al., 2018). Pakistan’s current IPC efforts (e.g., national guidelines, Lady Health Workers’ hygiene education) are steps in the right direction, but experience shows policy alone is insufficient. Strengthening IPC across income settings has been an explicit WHO priorit. This urban bias likely reflects the concentration of research institutions and laboratories in big cities. The implications of our findings are far-reaching. If Pakistan fails to aggressively contain infections and AMR, it not only faces a domestic health crisis where common infections become untreatable but also becomes a source of international spread. XDR typhoid and MDR TB from Pakistan cause outbreaks in other countries *via* travel (Saleem and Hassali, 2019; Khan et al., 2024). On the flip side, if Pakistan succeeds in bolstering WASH and IPC, it could serve as a model for other LMICs, showing that even with limited resources, strategic interventions can bend the curve of infections and AMR. Containing AMR in Pakistan would significantly contribute to global AMR containment given the country’s size. Crucially, Pakistan might leverage international support (the Fleming Fund, USAID’s Global Health Security Agenda, UNESCO, *etc.*, are already engaged) to accelerate implementation of surveillance and IPC training.

This review has several limitations. First, most included studies were observational or quasi-experimental in design, limiting causal inference regarding the effectiveness of IPC and WASH interventions on infection and AMR outcomes. Second, evidence was geographically concentrated in urban areas of Punjab and Sindh, with limited data from rural or underserved regions, potentially affecting generalizability. Third, heterogeneity in study quality, outcome measures, and reporting standards hindered quantitative synthesis. Fourth, data on antimicrobial resistance trends linked to IPC or WASH interventions were scarce, restricting deeper analysis of their direct impact on AMR. Fifth, as a narrative review, I did not perform a formal quality appraisal of each included study, which may introduce bias. The studies varied widely in design and rigor from randomized trials to single-center retrospective reports–and their reliability thus varies. Much of the grey literature lacked peer review, which may introduce reporting or policy bias. Finally, another limitation of this study is that the selection and synthesis of literature were conducted by a single author. While efforts were made to ensure a comprehensive and objective search through a hybrid methodology and the inclusion of official government documents, the lack of an independent second reviewer may introduce potential selection bias. Future updates to this status report could benefit from a multi-investigator panel to further enhance methodological triangulation. Despite these limitations, I believe the review provides a reasonably comprehensive overview of the landscape of infection preventive strategies in Pakistan with number of recommendations as described in Table 5. Moreover, there were few studies directly linked with IPC &WASH interventions to measure AMR outcomes, highlighting a critical research gap. Successful implementation of these strategies requires overcoming barriers like chronic underfunding, workforce shortages, and fragmented governance. Feasibility can be enhanced by leveraging existing community health networks for WASH education and adopting a phased, data-driven approach to IPC upgrades. Utilizing Public-Private Partnerships and international climate-health financing will be essential to meet resource requirements while ensuring that interventions are both sustainable and contextually appropriate for Pakistan’s diverse healthcare landscape. Future studies should integrate AMR surveillance with these interventions to provide stronger evidence on their impact on resistance patterns.

**TABLE 5:** Recommendations for policy, practice, and research.

Recommendation	Responsible stakeholders	Expected impact
1. Strengthen WASH infrastructure and hygiene	Government (Public Health Engineering, Local Govt), NGOs (WaterAid, *etc.*), Community leaders	Fewer infections (esp. diarrhea) – lowers antibiotic use in community; Long-term reduction in resistant enteric bacteria reservoir
2. Scale-up immunizations	EPI Programme (Ministry of Health), Provincial Health Depts	Prevention of pneumonia, typhoid, *etc.*, directly cuts down cases of drug-resistant infections (e.g., XDR typhoid) and need for antibiotics
3. Enforce IPC standards in hospitals (regular audits, IPC committees, funding for supplies)	Hospital administrations, Health regulatory authorities, Infection control professionals	Reduced HAIs and transmission of MDR organisms in facilities; improved patient outcomes. E.g., aiming to eliminate unsafe practices (open needle disposal, *etc.*)
4. Implement Antimicrobial Stewardship Programs (especially tertiary hospitals)	Hospital administrations, Pharmacies, Clinicians (ID physicians, microbiologists)	Optimized antibiotic use (stop overuse of broad-spec drugs); slower emergence of hospital-acquired resistance. Measured by lower consumption of reserved antibiotics and improved susceptibility patterns over time
5. Enhance diagnostic capacity and access (equip labs, subsidize cultures, introduce rapid tests)	Ministry of Health, Laboratory networks, Donors (Fleming Fund)	More accurate and faster pathogen identification enables de-escalation/targeted therapy. Should lead to decrease in empirical treatment failures and better patient management
6. Establish robust AMR surveillance network (national database, sentinel labs, annual reports)	NIH Pakistan (AMR surveillance cell), Provincial Public Health Labs, GLASS/WHO	Early warning of resistance trends (e.g., detection of new superbugs); data-driven policy adjustments. Fills current data gaps and measures intervention outcomes
7. Regulate and educate on antibiotic use (community) (enforce prescription-only law; public awareness campaigns)	Drug Regulatory Authority of Pakistan (DRAP), Pharmacy Council, Media, Medical associations	Curb over-the-counter antibiotic sales and self-medication leads to decreased inappropriate antibiotic consumption in the community. Better informed public will seek antibiotics less for viral illnesses
8. One Health approach	Ministry of Agriculture & Livestock, Veterinary Dept, Food Safety Authorities	Lower environmental Infection and AMR load; prevent transmission of resistant pathogens from animals to humans (e.g., resistant *Salmonella*, *E. coli*)
9. Support Infection and AMR research and innovation	HEC (Higher Education Commission), Research institutions, Funding agencies (WHO, Wellcome Trust, *etc.*)	New solutions tailored to local context. Evidence to inform policies (e.g., effective community messaging strategies). Builds capacity and keeps Pakistan on cutting-edge of AMR mitigation
10. Monitor and evaluate national action plan on AMR	National AMR Steering Committee, Independent evaluators	Accountability and sustained momentum. Helps identify what’s working vs. not, allowing course-corrections. International credibility

## Conclusion

This review highlights that combating infectious diseases and AMR in Pakistan requires a multifaceted, prevention-focused approach. The evidence summarized here provides a valuable foundation to guide policymakers, healthcare professionals, and communities in driving this critical transformation. Although the challenges are considerable, they are not insurmountable. Breaking the chain of infection will take time, but integrated, evidence-based strategies across healthcare and community settings can markedly reduce the infectious disease burden. Achieving lasting progress toward national and global AMR goals will depend on sustained investment, behavioral change, and effective policy implementation.

## Data Availability

The original contributions presented in the study are included in the article/supplementary material, further inquiries can be directed to the corresponding author.
